# Mitigating Interobserver Variability in Radiomics with ComBat: A Feasibility Study

**DOI:** 10.3390/jimaging10110270

**Published:** 2024-10-24

**Authors:** Alessia D’Anna, Giuseppe Stella, Anna Maria Gueli, Carmelo Marino, Alfredo Pulvirenti

**Affiliations:** 1 Department of Physics and Astronomy “E. Majorana”, University of Catania, Via Santa Sofia 64, 95123 Catania, Italy; alessia.danna@phd.unict.it (A.D.); anna.gueli@unict.it (A.M.G.); 2 Department of Medical Phyisics, Humanitas Istituto Clinico Catanese (H-ICC), Contrada Cubba S.P. 54 n.11, 95045 Misterbianco, Italy; carmelo.marino@humanitascatania.it; 3 Department of Clinical and Experimental Medicine, University of Catania, Via Santa Sofia 64, 95123 Catania, Italy; alfredo.pulvirenti@unict.it

**Keywords:** radiomics, multicenter studies, precision medicine, clinical imaging, segmentation, batch correction

## Abstract

This study investigates Intraobserver Features Variability (IFV) in radiomics studies and assesses the effectiveness of the ComBat harmonization method in mitigating these effects. *Methods:* This study utilizes data from the NSCLC-Radiomics-Interobserver1 dataset, comprising CT scans of 22 Non-Small Cell Lung Cancer (NSCLC) patients, with multiple Gross Tumor Volume (GTV) delineations performed by five radiation oncologists. Segmentation was completed manually (“vis”) or by autosegmentation with manual editing (“auto”). A total of 1229 radiomic features were extracted from each GTV, segmentation method, and oncologist. Features extracted included first order, shape, GLCM, GLRLM, GLSZM, and GLDM from original, wavelet-filtered, and LoG-filtered images. *Results:* Before implementing ComBat harmonization, 83% of features exhibited *p*-values below 0.05 in the “vis” approach; this percentage decreased to 34% post-harmonization. Similarly, for the “auto” approach, 75% of features demonstrated statistical significance prior to ComBat, but this figure declined to 33% after its application. Among a subset of three expert radiation oncologists, percentages changed from 77% to 25% for “vis” contouring and from 64% to 23% for “auto” contouring. This study demonstrates that ComBat harmonization could effectively reduce IFV, enhancing the feasibility of multicenter radiomics studies. It also highlights the significant impact of physician experience on radiomics analysis outcomes.

## 1. Introduction

Over the past few years there has been an increase of interest in radiomics [1,2,3,4,5]. Quantitative imaging enables various prediction and classification tasks to be addressed and, alongside other -omics sciences, contributes to the advancement of precision medicine.

For this purpose, radiomics-based analyses should be as generalizable as possible and supported by larger and more diverse datasets, allowing for more robust inference [6,7,8]. However, to date, the majority of studies have been conducted within a single institution, and radiomics models are frequently constructed using small datasets without external validation. Multicenter studies could be a possibility, especially with the advent of federate learning [9,10], a solution for data-private multi-institutional collaborations. In this approach, model-learning leverages all available data without sharing data among institutions, distributing model training to data owners, and aggregating their results.

One challenge in this scenario arises from variations in imaging protocols across different institutions. These differences encompass acquisition methods, post-processing of images, and reconstruction techniques [6,11,12,13,14,15]. Hence, these discrepancies may affect radiomic features, leading to not robust models, and hiding existing correlations or finding non-existent ones.

Given that numerous radiomic features are highly sensitive to these factors, it becomes a troubleshooting issue to reconcile these discrepancies. To tackle it, various standardization approaches have been proposed, which can be categorized into two main groups: image-based or feature-based methods [6,16]. Further information can be found in Da-Ano et al. [6] and Horng et al. [16]. Feature-based methods include the selection of robust features in connection with batch correction methods [6,16], where batch effects are non-biological factors that affects data, e.g., experimental settings (scanner, protocols) and observer variability. Unlike feature selection methods, batch effect correction methods effectively standardize data post-feature extraction without further loss of information [16].

Among batch correction methods, ComBat (Combating Batch effects) stands out as one of the most promising ones [17,18,19,20,21,22]. Chen et al. [18] and Fortin et al. [23] have found that ComBat consistently outperformed other adjustment methods and is the most robust approach in the case of small datasets.

Despite the fact it was originally designed for genomics [17], ComBat has been used for radiomics to pool images from different sites or scanners, thus overcoming the lack of data in radiomics studies. Since 2017, at least 51 papers have reported the use of ComBat in radiomic analysis of Magnetic Resonance (MR) (36%), Computed Tomography (CT) (34%), or Positron Emission Tomography (PET) images (28%) [20].

Among them, Orlhac et al. [22] investigated whether a compensation method could correct for the variations of radiomic feature values caused by using different CT protocols. The correction for scanner effect was confirmed in patient data with 100% (10 of 10 features for phantom CT scans) and 98% (87 of 89 features for patients CT scans) of *p*-values less than 0.05 before correction, compared with 30% (three of 10) and 15% (13 of 89) after correction.

Foy et al. [24] investigated the effects of CT image acquisition and reconstruction parameters using a cadaveric liver and determined harmonization methods to mitigate these variations. Histogram normalization reduced or maintained the number of significantly different features for all scans, while ComBat reduced the number of significantly different features to zero for all scans.

In another study [21], authors investigated the use of ComBat for MR radiomics finding that, in brain tumors, 41 (FLAIR) or 36 (CE-T1w) out of 42 features were significantly different between the 1.5- and 3-T images without harmonization, against 1 (FLAIR) or none (CE-T1w) with harmonization. In prostate studies the ability to distinguish between Gleason Grades (GGs) using radiomic features was increased after ComBat: 636 radiomic features were significantly different GGs between after harmonization against 461 before. Saint Martin et al. [25] proposed a radiomics pipeline dedicated to breast MR images which includes ComBat harmonization; they found that ComBat lowered the percentage of radiomic features significantly different from 87%.

In these and other works [22], ComBat has enabled the harmonization of features, thereby enhancing statistical analysis. Given the successes of these studies, we have chosen to investigate the impact of a batch effect that could potentially influence both multi-institutional and single-institutional studies, i.e., the interobserver variability.

When we talk about radiomics, a typical workflow includes key stages such as image acquisition and segmentation, followed by the extraction of radiomics features, statistical analysis, and the development of diagnostic and prognostic models [26].

One of the first steps in this workflow is the segmentation of the Volume of Interest (VOI), from which features are extracted. It is usually performed manually by radiation experienced oncologists with good expertise, but different operators often produce variable results. Inconsistencies in segmentations have been reported for both inter- and intraobserver contours variability [27,28,29,30,31].

These discrepancies arise from the subjective nature of this step: the expert conducting the segmentations evaluates the available images and subsequently decides, drawing from prior knowledge and experience, which voxels to include in the VOI. This variability could have a significant impact on quantitative analysis. Notably, the assessment of observer variability as a batch effect has not, to our knowledge, been previously explored in the context of radiomic features for CT scans. Following the assessment of this source of variability in the extracted features, we examined the effectiveness of ComBat in reducing IFV. Our analysis was conducted using a public dataset, specifically the NSCLC-Radiomics-Interobserver1 dataset [32,33,34,35] available on The Cancer Imaging Archive (TCIA) [33].

## 2. Materials and Methods

### 2.1. Dataset Description

NSCLC-Radiomics-Interobserver1 [32,33,34,35] contains clinical data and from PET confirmed not metastatic Non-Small Cell Lung Cancer (NSCLC) radiotherapy patients. It comprises 22 individuals, with 9 females (41%) and 13 males (59%). The patients’ average age is 64 years, ranging from 40 to 82 years.

The dataset includes various types of lung tumors, such as adenocarcinoma, squamous cell carcinoma, large cell carcinoma, and undifferentiated lung carcinoma, with the specific type varying depending on the patient. T stage, which characterizes the size and extent of the primary tumor, ranges from T1 to T4 (except for T3), indicating the degree of invasion into nearby tissues. The N stage signifies the involvement of nearby (regional) lymph nodes, with values ranging from 0 to 3, indicating the extent of lymph node involvement. The M stage, which assesses the presence of distant metastasis, is consistently recorded as 0, indicating the absence of distant metastasis in all cases. Both images and clinical data are accessible on TCIA [33].

Pre-treatment CT scans used for radiotherapy planning (spiral CT scans of the whole thorax with intravenous contrast) are available for each patient. For 20 of these patients (excluding patients 9 and 19), segmentations of the 3D volume of the Gross Tumor Volume (GTV) on CT scans were performed by five different radiation oncologists. This was achieved through two approaches: blinded manual delineation (“vis”) and the use of an in-house autosegmentation tool for the initial delineation, followed by manual adjustment of the primary GTV outline (“auto”). For some patients, two different volumes were present, labeled as GTV-1 and GTV-2. However, GTV-2 segmentations were specifically associated with the mediastinal region and were only available for a subset of patients and radiation oncologists. Due to this limited availability, these GTV-2 segmentations were deemed ineligible for inclusion in this study and were consequently excluded from the analysis.

**Figure 1:**
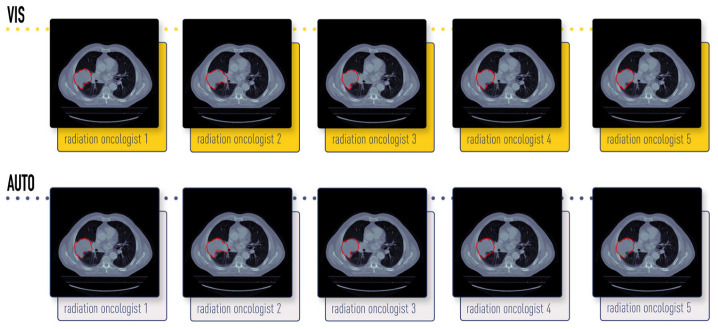
Example of “vis” and “auto” GTV-1 contouring (patient 8) for each radiation oncologist. NSCLC-Radiomics-Interobserver1 dataset [32,33,34,35].

The radiation oncologists involved had different degrees of experience: ‘1’ and ‘3’ were trainee radiation oncologists at the time of this experiment, while ‘2’, ‘4’, and ‘5’ were extensively experienced. Figure 1 provides visual examples of both contouring approaches by each radiation oncologist for patient 8. Metadata report a single Siemens (Siemens Healthcare, Forchheim, Germany) scan model.

### 2.2. Features Extraction

DICOM (Digital Imaging and COmmunications in Medicine) series were converted into NRRD (Nearly Raw Raster Data) format. Considering the exploratory nature of this study, we chose to extract the features indicated in the example .yaml file for CT in the PyRadiomics documentation.

Images were employed in three different modalities: their original unaltered form, subjected to Laplacian of Gaussian (LoG) filtering with different sigma values (σ = [2.0, 3.0, 4.0, 5.0]) to reflect fine (2.0), medium (3.0, 4.0), and coarse textures (5.0), and subjected to wavelet-based filtering with eight different decompositions [high-low-low (HLL), low-high-low (LHL), low-high-high (LHH), low-low-high (LLH), high-low-high (HLH), high-high-high (HHH), high-high-low (HHL), and low-low-low (LLL)]. The coif1 wavelet package (PyWavelets library, v0.4.0) was used to generate wavelet images.

Feature extraction encompassed shape, first order, Gray-Level Co-occurrence Matrix (GLCM), Gray-Level Run-Length Matrix (GLRLM), Gray-Level Size Zone Matrix (GLSZM), and Gray-Level Dependence Matrix (GLDM) features were extracted. The extraction process was executed using the precision-medicine-toolbox Python package (version 0.11) [36]. This package facilitated the preparation of imaging datasets and the comprehensive exploration of their associated features. The precision-medicine-toolbox relies on PyRadiomics, with most of its features adhering to the definitions established by the Imaging Biomarker Standardization Initiative (IBSI) [37].

**Figure 2:**
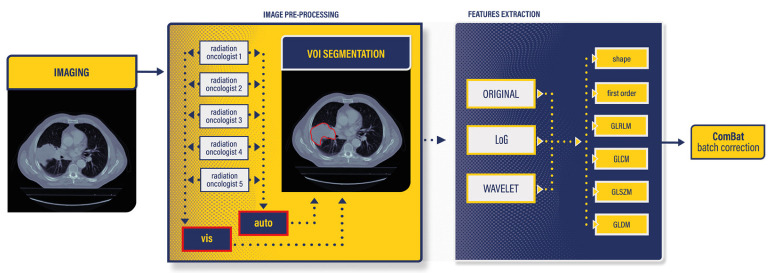
Radiomics workflow. CT images contoured by five different radiation oncologists, employing two distinct approaches: “vis” and “auto”. Feature extraction encompassed both original, wavelet-filtered, and LoG-filtered images, incorporating shape, first order, Gray-Level Co-occurrence Matrix (GLCM), Gray-Level Run-Length Matrix (GLRLM), Gray-Level Size Zone Matrix (GLSZM), and Gray-Level Dependence Matrix (GLDM) features. A post-extraction ComBat correction was conducted to reduce Interobserver Features Variability (IFV).

Feature extraction was carried out individually for each radiation oncologist, for image types (original, LoG, filtered, and wavelet-filtered), and contouring method (“vis” and “auto”). The radiomics workflow, spanning from CT imaging to batch correction, is visually summarized in Figure 2; at the end of this process 1229 features were extracted for each image.

### 2.3. Features Harmonization

ComBat estimates scanner-specific location and scale parameters, for each feature separately, and pools information across features using empirical Bayes to improve small sample size studies. It starts from the following assumption:

y_ij_ = α + γ_i_ + δ_i_ϵ_ij_ (1)

where *j* indicates the specific measurement of the *y-feature*, α is the average value feature *y*, γ_i is an additive batch effect that affect the measurement, δ_i is a multiplicative batch effect, and ϵ_ij is an error term. Additionally, *i* indicates the batch, i.e., the experimental setting for the *y* measurement which includes scanner effects and, as in our specific case, possible observer effects. These effects can be corrected using the realignment transformation expression:

(2) $${y_{ij}}^{ComBat}=(y_{ij}-\hat{\alpha }-{\hat{\gamma }}_{i})/{\hat{\delta }}_{i}+\hat{\alpha }$$

where $\hat{\alpha }$, ${\hat{\gamma }}_{i}$, and ${\hat{\delta }}_{i}$ are estimators respectively of α, γ_i_, and δ_i_, and y_ij_^ComBat^ is the y_ij_ value corrected for site effects. Equation (2) is the ComBat simplest expression; for distributions itself composed of two or more distributions, a covariate is needed. In this study datasets differ only for the oncologist which perform the tumor contouring, so a covariate is not necessary.

ComBat is a data-driven method and for this reason, harmonization must be carried out for each tissue or tumor type or patient population. Batch correction is also feature-specific because different features are affected in different ways by site effects. This kind of harmonization can be applied in two different ways, i.e., realigning distributions to a virtual site or by choosing one as a reference. Although the absolute value of features changes, both of these approaches lead to the same results: identical ROC curves for classification tasks are obtained [20]. Considering that, the first approach was chosen for this study. Harmonization was performed by making use of the neuroCombat (0.2.12) python package [23]. Despite the small dataset size, Orlhac et al. [20] found that the results support the recommendation of using ComBat when at least 20–30 patients per batch are available, particularly when no covariate is included, as is the case in our study.

A statistical analysis was conducted to assess the impact of contouring, denoted as the independent variable *i* in Equation (1), on the distributions of radiomic feature values, represented as dependent variables *y_ij_* in Equation (1). For this purpose, we employed two-sided Friedman tests both before and after applying ComBat harmonization to each feature. The Friedman test was conducted using the *friedman.test* function from the stats package in R 4.1.2 (R Core Team, 2021), considering the patients as blocks and the radiation oncologists as groups. To maintain the desired level of statistical rigor, we employed the Benjamini–Hochberg procedure to control the false discovery rate. The statistical test was repeated, focusing exclusively on experienced radiation oncologists, with the aim of investigating whether clinician experience could be considered a significant factor in radiomics studies.

## 3. Results

**Figure 3:**
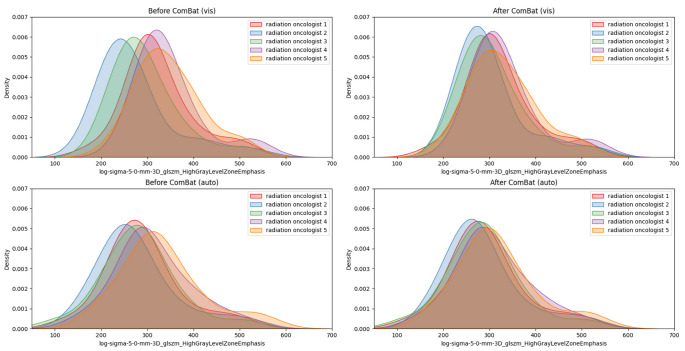
Density plots illustrating the distribution of “log-sigma-5-0-mm-3D_glszm_HighGrayLevelZoneEmphasis” before (left) and after (right) applying ComBat for the “vis” (upper) and “auto” (lower) contouring approaches. The data are segregated for five radiation oncologists: radiation oncologist 1 (in red), 2 (in blue), 3 (in green), 4 (in purple), and 5 (in orange). The data are aligned on a virtual site for comparison, showcasing changes in the feature’s distribution due to the ComBat transformation.

The primary objective of the harmonization process was to align the features distributions concerning mean and standard deviation. Figure 3 illustrates the effect of the ComBat method on the density plot for a chosen example feature (log-sigma-5-0-mm-3D_glszm_HighGrayLevelZoneEmphasis), contoured by each radiation oncologist. Before employing ComBat, the *p*-value stood at 6.03 × 10^−5^, but following the harmonization step, specifically the “vis” contouring, it increased to 8.0 × 10^−2^. Similarly, for the “auto” approach, the *p*-value shifted from 2 × 10^−3^ to 1.7 × 10^−1^. After ComBat, the feature distributions were aligned with a virtual site. Comparable results were observed for the other features and contouring approaches.

A *p*-value exceeding 0.05 in the Friedman test indicated the successful achievement of distribution realignment. In the case of “vis” contouring, the analysis revealed a significant shift in feature significance levels. Prior to applying ComBat, 83% (1018 of 1229) of the features exhibited *p*-values below the chosen threshold, while after harmonization, this percentage reduced to 34% (413 of 1229). Also, for “auto” contouring, a remarkable change was observed, with 75% (902 of 1229) of features showing significance before compensation, which decreased to a mere 33% (404 of 1229) after applying ComBat.

**Figure 4:**
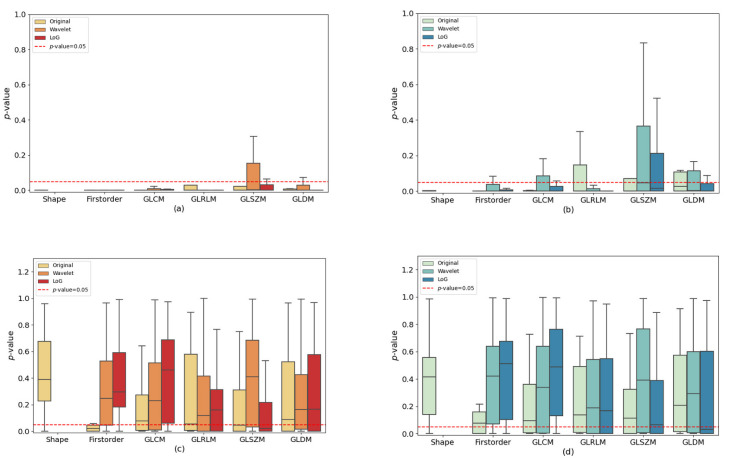
Whole sample (radiation oncologists 1–5) *p*-value boxplots. Comparison of radiomic feature percentages, categorized by image type (original, wavelet, LoG) and feature classes (Shape, first order, GLCM, GLRLM, GLSZM, GLDM), alongside *p*-values obtained from the Friedman test (Benjamini–Hochberg corrected). The dashed red line represents the significance threshold (*p*-value = 0.05). Panels (**a**,**b**) display the data before ComBat normalization, while panels (**c**,**d**) showcase the data after normalization. The “vis” segmentation approach is depicted in a red color scale, while the “auto” segmentation approach is represented with a blue color scale.

**Figure 5:**
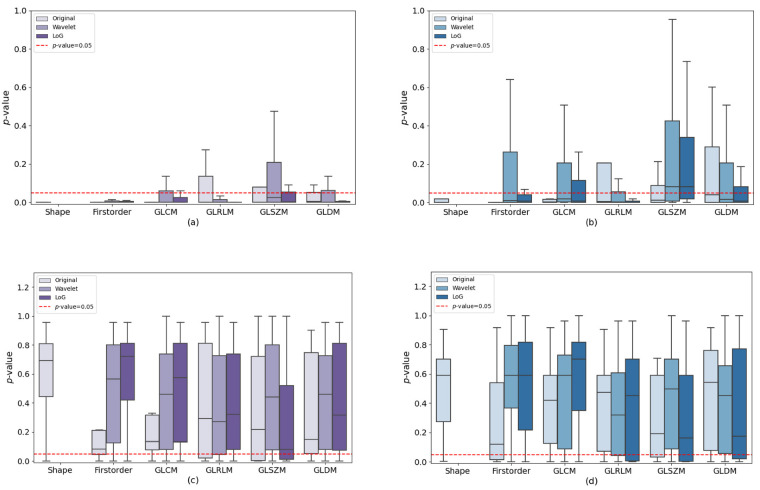
Expert radiation oncologist (radiation oncologist 2, 4, and 5) *p*-value boxplots. Comparison of radiomic feature percentages, categorized by image type (original, wavelet, LoG) and feature classes (Shape, first order, GLCM, GLRLM, GLSZM, GLDM), alongside *p*-values obtained from the Friedman test (Benjamini–Hochberg corrected). The dashed red line represents the significance threshold (*p*-value = 0.05). Panels (**a**,**b**) display the data before ComBat normalization, while panels (**c**,**d**) showcase the data after normalization. The “vis” segmentation approach is depicted in a purple color scale, while the “auto” segmentation approach is represented with a grey color scale.

Notably, when considering only the subset of three expert radiation oncologists, the percentages shifted from 77% (951 of 1229) before to 25% (276 of 1229) after ComBat for “vis” contouring, and from 64% (786 of 1229) to 23% (287 of 1229) for “auto” contouring. Results for all radiation oncologists are summarized in Figure 4 divided for image type, feature classes, and segmentation approach. Figure 5 shows *p*-values boxplots only for experienced radiation oncologists.

## 4. Discussion

In this study, we explored interobserver variability in radiomics and assessed the efficacy of ComBat as a method to mitigate this batch effect across different datasets. This harmonization step could potentially enhance the performance of prediction and classification models in personalized medicine by reducing spurious correlations between radiomic features and underlying biology. Notably, in the dataset used, while the identified GTV on the same CT scan remains consistent, there are evident variations in contouring practices among different radiation oncologists (Figure 1). Some of them tend to delineate wider or narrower contours, resulting in distinct VOIs from which radiomic features are extracted. This variance in segmentation leads to different data to correlate with clinical outcomes for the same patient.

The outcomes derived from the Friedman test provide valuable insights into the stability of radiomic features. T 4 displays boxplots of *p*-values for each radiation oncologist, image type, and feature class, both before and after applying ComBat normalization. When considering “vis” contouring, our analysis highlights that, prior to applying ComBat, GLSZM features demonstrate a degree of robustness to contouring variations only if used in conjunction with the wavelet filter. All other features exhibit *p*-values < 0.05 (excluding outliers). Considering the same segmentation approach, original and LoG-filtered images, all features demonstrate *p*-values consistently below 0.05 for all classes of radiomic features (except for outliers).

When employing the “auto” approach, certain GLRLM, GLSZM, and GLDM features display *p*-values exceeding 0.05 before applying ComBat to original images. Interestingly, features extracted from wavelet-filtered images demonstrate greater stability in comparison to other image types, particularly for GLCM, GLSZM, and GLDM. GLSZM features also demonstrate similar outcomes in the case of LoG-filtered images. In this scenario, features extracted from the original images appear to be the most sensitive to contouring variations, even though some of them have *p*-values greater than 0.05. Following the application of ComBat, the majority of *p*-values exceed the 0.05 threshold, except for first-order features in original images.

Notably, the most challenging results are observed for first order features extracted from original images, while the most favorable outcomes are observed for shape features extracted from original images. These results demonstrate the sensitivity of radiomic features to contouring discrepancies, underlining ComBat’s effectiveness in mitigating inter-feature variation (IFV).

Regarding the contouring approaches, a slightly greater IFV was noted for the “vis” segmentation (about the 10%). This result is also evident from the density plot in Figure 2 Moreover, this observation is reinforced by the findings of Kothari et al. [38], who reported a higher Dice Coefficient (DC) for the “auto” contouring in comparison to the “vis” contouring. This is likely due to the fact that in the case of semiautomatic contouring, radiation oncologists receive the same suggestion from the tool and simply make adjustments to it.

However, consistent results were achieved after applying ComBat to both contouring methods. This observation highlights that harmonization effectively addresses the collective variances stemming from differences in contouring techniques and interobserver disparities. Moreover, this discovery holds true when examining experienced radiation oncologists, where their expertise proves to be a substantial influencing factor. Among these seasoned professionals, only 23% and 25% (as illustrated in Figure 5) of the characteristics yielded *p*-values below 0.05 for “vis” and “auto,” respectively. This is in contrast to the percentages of 34% and 33% observed within the entire dataset.

Standardizing data has become a focal point in the global imaging community, with growing recognition of the importance to mitigate variations in radiomic features across different centers and imaging machines. ComBat has proven to be a valuable tool for reducing batch effects and as revealed by this study, seems to be a good solution to reduce IFV. One of its distinguishing features is its user-friendly nature, as it can be applied a posteriori without necessitating any modifications to the original images.

However, despite being simple to use, it has some limitations. First of all, ComBat can only harmonize a single batch effect at time. Additionally, as underlined from Horng et al. [16], ComBat assumes that all batch effects and clinical covariates are known before the statistical analysis, but this is not always true. Consequently, some variations may be introduced due to information not included in the dataset. Another assumption is that errors from standardized input data follow a normal distribution, but in some cases, feature can have multimodal distributions [16].

Regarding the specific use of ComBat proposed in this study for mitigating IFV, it is important to note that in its current form, it is applicable only to single center studies. For multicenter studies, it could be included as a covariance factor, although further research is necessary to confirm its feasibility. Despite these limitations, obtained results show that by complying with the application conditions of ComBat, it succeeds in reducing IFV. Fully automatic segmentation methods can be another solution (e.g., Convolutional Neural Networks); however, these approaches have some limitations due to inhomogeneous density and unclear boundaries, especially for tumors adjacent to structures with similar densities. Moreover, at the present time these methods are not routinely used in clinics.

## 5. Conclusions

This study explored the use of ComBat for reducing interobserver variability in radiomic features. ComBat is a data-driven method, so it can be directly applied to extracted features and thus does not require additional image processing. This makes it useful for both single- and multi-institutional studies and for resolving the problem of data scarcity in rare diseases or pediatric patients. The research revealed that ComBat could potentially prove effective in reducing Interobserver Features Variability (IFV) for both manual contouring and using an in-house autosegmentation tool followed by manual adjustment. We also found that radiation oncologist experience has a high impact on radiomics analysis quality. Despite its limitations, ComBat can be a useful tool for reducing IFV, particularly in the absence of automatic or semiautomatic contouring methods. The current interest in clinical data sharing through Federated Learning makes ComBat even more useful for future radiomics studies.

## Data Availability

Data are available at https://www.cancerimagingarchive.net/collection/nsclc-radiomics-interobserver1/ (accessed on 15 January 2023).
